# PCL and PCL/bioactive glass biomaterials as carriers for biologically active polyphenolic compounds: Comprehensive physicochemical and biological evaluation

**DOI:** 10.1016/j.bioactmat.2020.11.025

**Published:** 2020-12-05

**Authors:** Michal Dziadek, Kinga Dziadek, Kamila Checinska, Barbara Zagrajczuk, Monika Golda-Cepa, Monika Brzychczy-Wloch, Elzbieta Menaszek, Aneta Kopec, Katarzyna Cholewa-Kowalska

**Affiliations:** a Jagiellonian University, Faculty of Chemistry, 2 Gronostajowa St., 30-387, Krakow, Poland; b AGH University of Science and Technology, Faculty of Materials Science and Ceramics, Department of Glass Technology and Amorphous Coatings, 30 Mickiewicza Ave., 30-059, Krakow, Poland; c University of Agriculture in Krakow, Faculty of Food Technology, Department of Human Nutrition and Dietetics, 122 Balicka St., 30-149, Krakow, Poland; d Jagiellonian University, Medical College, Department of Molecular Medical Microbiology, 18 Czysta St., 31-121, Krakow, Poland; e Jagiellonian University, Medical College, Department of Cytobiology, 9 Medyczna St., 30-688, Krakow, Poland

**Keywords:** Tissue engineering, Drug delivery, Antioxidant properties, Plant extract

## Abstract

In this work, polymeric and bioactive glass (BG)-modified composite films were successfully loaded with polyphenols (PPh) extracted from sage. It was hypothesized that PPh, alone and in combination with BGs particles, would affect physicochemical and biological properties of the films. Furthermore, sol-gel-derived BG particles would serve as an agent for control the release of the polyphenolic compounds, and other important properties related to the presence of PPh. The results showed that polyphenolic compounds significantly modified numerous material properties and also acted as biologically active substances. On the one hand, PPh can be considered as plasticizers for PCL, on the other hand, they can act as coupling agent in composite materials, improving their mechanical performance. The presence of PPh in materials improved their hydrophilicity and apatite-forming ability, and also provided antioxidant activity. What is important is that the aforementioned properties and kinetics of PPh release can be modulated by the use of various concentrations of PPh, and by the modification of PCL matrix with sol-gel-derived BG particles, capable of binding PPh. The films containing the lowest concentration of PPh exhibited cytocompatibility, significantly increased alkaline phosphatase activity and the expression of bone extracellular matrix proteins (osteocalcin and osteopontin) in human normal osteoblasts, while they reduced intracellular reactive oxygen species production in macrophages. Furthermore, materials loaded with PPh showed antibiofilm properties against Gram positive and Gram negative bacteria. The results suggest that obtained materials represent potential multifunctional biomaterials for bone tissue engineering with a wide range of tunable properties.

## Highlights

• PPh-loaded films showed improved hydrophilicity and calcium phosphate-forming ability.
• The presence of PPh in the films improved mechanical properties and provided antioxidant activity.
• PPh release kinetics can be modulated by the use of sol-gel-derived BG particles as PCL modifier.
• Materials enriched with PPh showed antibiofilm properties against Gram‐positive and Gram‐negative bacteria.
• Materials enriched with PPh showed anti-inflammatory properties and enhanced osteostimulative activity.

## Graphical abstract

**Figure:**
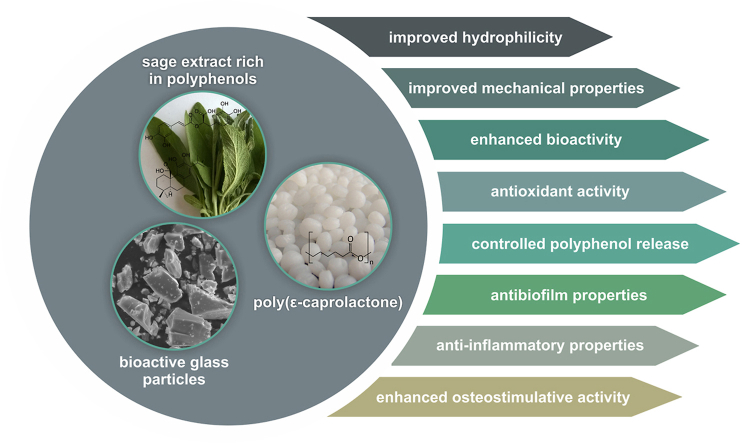

## 1 Introduction

Over the last few years, the search for new strategies for bone tissue reconstruction has become one of the priorities of medical research. The necessity of obtaining novel bone substitutes is driven by functional disabilities of patients and aesthetic defects with far-reaching psychosocial implications [1]. The current problem of treatment hard tissue defects is a growing medical and socioeconomic challenge for the ageing population of developed countries. An attractive alternative for the therapeutic protocols implemented so far, including autogenous, allogenic and xenogenic transplants, is bone tissue engineering (BTE), which invents even more effective methods of controlled tissue regeneration [2]. However, the use of biomaterials often entails the risk of frequent bacterial infections which can cause numerous complications at the local or even systemic level [3]. Another problem associated with tissue damage and biomaterial implantation is an inflammatory response. While the acute phase of inflammation is essential for proper tissue regeneration, the chronic inflammatory process may lead to delayed or unsuccessful recovery [4,5]. What is more, a certain percentage of bone defects results from intentional resection procedures that are part of cancer treatment. In this case, a major problem is cancer recurrence that arises from residual cancer cells [6].

The main features of the biomaterials used in BTE are osteoconductivity and osteoinductivity. Particularly well-known for these properties are bioactive glasses (BGs). BGs are characterized by their ability to form a direct chemical bond with bone at the implantation site through the mineralization of a biomimetic apatite layer [7]. Furthermore, the dissolution products, especially silica and calcium ions, stimulate osteogenesis and new bone formation [8]. What is important, sol-gel-derived BGs, because of their high surface area and the presence of siloxane groups (Si–OH) in their structure, show enhanced activity in biological environment in comparison with conventional melt-derived glasses [7,9].

Taking into account the aforementioned problems, biomaterials with new levels of biofunctionality, that can meet complex requirements of BTE, should be designed. Bioactive materials with osteostimulative properties can be improved by introducing additional activities such as antibacterial, anti-inflammatory, anti-cancerous, and antioxidant properties. For this purpose, numerous active substances are used to modulate the response of the human body's environment. BTE scaffolds are used as carriers of active substances, such as antibiotics (e.g. gentamycin, vancomycin, tetracycline, gatifloxacin); anti-inflammatory drugs (e.g. dexamethasone, ibuprofen); as well as anti-cancer drugs (e.g. doxorubicin, methotrexate, cisplatin) [10,11]. Despite significant improvements in active substances bioavailability and avoidance of side effects associated with systemic drug administration, there are still some limitations regarding their usage in local delivery systems. One of the growing concerns is the increasing prevalence of antibiotic-resistant bacteria [3]. Moreover, it had been shown that bactericidal antibiotics are toxic to mammalian cells in direct contact, causing oxidative damage to DNA, proteins, membrane lipids, and also mitochondrial dysfunction [12]. In cancer treatment, a major problem in the use of chemotherapeutic agents is high toxicity of these drugs to normal cells and healthy tissues. On the other hand, the effectiveness of conventional chemotherapeutics is limited by drug resistance, especially in the case of bone tumors (e.g. osteosarcoma, Ewing's sarcoma, metastatic tumors) [13,14]. Furthermore, the efficacy of chemotherapeutic agents is dramatically reduced due to their unfavorable physicochemical and pharmacological properties, including poor water affinity [15]. All these features indicate that the currently available solutions are not without flaws and require a new, innovative approach.

In the last decades, many efforts have been made to overcome the above-mentioned drawbacks by using alternative active substances, i.a. compounds produced by plants – phytochemicals. In the group of plant-derived compounds with beneficial effects on human health, polyphenols, in particular, are under investigation as therapeutic agents due to their multi-faceted activity. In the literature there are reports of polyphenols high antiproliferative activity towards cancer cell lines (including melanoma, lung, liver, and prostate cancer), as well as of their antimicrobial and anti-inflammatory activity [[16], [17], [18]]. Moreover, numerous studies indicate that extracts from medical sage (*Salvia officinalis* L.) are rich in a variety of polyphenolic compounds, promising for use in biomedical applications, such as flavonoids and phenolic acids, whose concentration in sage is higher compared to other herbs [19]. Attention should be paid to the controlled release of polyphenols to achieve the expected therapeutic effect.

The use of BG particles as modifiers of poly(ε-caprolactone) (PCL) matrix has been widely reported in the literature. This approach allows for obtaining bioactive composites with osteostimulative properties and improved mechanical behavior [20,21]. Furthermore, BG particles have been shown to modify PCL matrix wettability, and therefore degradation rate and cell response [20]. On the other hand, PCL-based materials have been enriched with phytochemicals to improve their biological properties. PCL nanofibers loaded with caffeic acid and (−)–Epigallocatechin–3–O–gallate (EGCG) have exhibited strong anticancer activity against human cancer cells [22]. Jain et al. [23] and Venugopal et al. [24] have shown that incorporation of curcumin and phytochemicals extracted from *Wattakaka volubilis* in PCL nanofibers enhanced osteoblastic cell functions. In turn, PCL scaffolds enriched with *Areca catechu* extract have been characterized by antioxidant, antimicrobial, and wound-healing properties [25].

In the literature, there is limited data on combination of PCL, BG and phytochemicals. Our recent studies have indicated that PCL/BG composites enriched with polyphenols extracted from sage, as well as fruits and leaves of sweet cherry represent potential multifunctional biomaterials for BTE [26,27]. However, in the aforementioned works, polyphenols were introduced into materials directly with solvent-plant extract, which limits the possibility of obtaining a precise final concentration of polyphenols in composites. In this work, solid sage extract was obtained and used. PCL and PCL/BG films enriched with different concentrations of polyphenolic compounds (1.5, 3, and 4.5% w/w) were designed and developed. Sol-gel derived BG particles were used as a modifier in composites. The films were comprehensively evaluated in terms of surface properties, including microstructure, wettability, and surface energy (i); mechanical properties (ii); structural properties, including the degree of crystallinity and crystallite size (iii); *in vitro* apatite-forming ability (iv); *in vitro* release of polyphenols and antioxidant activity (iv); cytotoxicity, *in vitro* osteogenic and anti-inflammatory properties (v); as well as antibacterial activity against *Staphylococcus aureus* and *Pseudomonas aeruginosa* (vi). It was hypothesized that the polyphenols, alone and in combination with bioactive glass particles would affect the listed above properties. Furthermore, BG particles would serve as an agent for controlling not only the release of the polyphenolic compounds but also other important properties related to the presence of polyphenols.

## 2 Materials and methods

### 2.1 Plant extract preparation

Solvent extraction was performed in 1,4-dioxane (Avantor Performance Materials, Poland)/deionized water mixture (4:1 vol ratio) with the solid-liquid ratio of 1:20 (w/v). Leaves of sage (*Salvia officinalis* L.) were randomly collected from plants grown in the experimental garden (the University of Agriculture in Krakow). Lyophilized leaves of sage were ground in the mortar and mixed with a solvent in Erlenmeyer flask. Extraction was carried out by shaking at room temperature in the dark for 24 h. Then the extract was filtered through filter paper under vacuum and centrifuged (1500 rpm, 15 min). To obtain a solid extract, the solvent was removed from liquid extract using rotary an evaporator (Rotavapor R-300, Buchi, Switzerland) followed by lyophilization (Alpha 1–4, Christ Martin, Germany). The obtained solid extract was stored at −20 °C for further use.

### 2.2 Material preparation

Bioactive glass with the composition of (mol%) 40SiO_2_–54CaO–6P_2_O_5_ (A2) were prepared using the sol-gel method described previously [28]. All the reagents used for BG synthesis were purchased from Sigma-Aldrich, Germany. BG was milled to obtain the powder with a particle size of 1.5 μm (d_50_).

The films were prepared using the solvent casting method. Firstly, the solid plant extract was dissolved in 1,4-dioxane/deionized water mixture (4:0.3 vol ratio) in an ultrasonic bath for 5 min. To obtain composite films, BG particles were mixed with extract solution and stirred for 24 h with a magnetic stirrer. Afterwards, poly(ε-caprolactone) pellets (PCL, Sigma-Aldrich, Germany, M_n_ = 80 kDa, M_w_/M_n_ < 2) were added to the BG suspension and stirred for the next 24 h. The content of BG in composites was 30% w/w, while the concentrations of PPh were 1.5, 3, and 4.5% w/w. The polymer concentration in prepared solutions was 10% w/v. The suspension was cast onto glass Petri dishes (diameter 90 mm), which were covered with glass Petri dish lids (diameter 100 mm). The drying process was carried out at 40 °C for 7 days followed by drying under vacuum at ambient temperature for 24 h. The polymer films were prepared in the same way, excluding the BG addition stage. The materials were stored at −20 °C until further investigation. The surfaces of obtained films that have been exposed to the solvent vapor during casting were denoted as AS, while the surfaces that have been in contact with the glass Petri dish were marked as GS.

### 2.3 Material evaluation

#### 2.3.1 Evaluation of the surface properties

Microstructure and chemical composition were analyzed with scanning electron microscopy (SEM, Nova NanoSEM 200, FEI, Netherlands) coupled with energy dispersion X-ray analyzer (EDX, EDAX, Netherlands). The samples were coated with a carbon layer before evaluation. Surface wettability and solid-state surface energy were evaluated using a sessile drop method with an automatic drop shape analysis system DSA 25 (Kruss, Germany). Ultra-high-quality water (UHQ, PureLab, Vivendi Water) and diiodomethane (Sigma-Aldrich, Germany) were used to determine the polar-dispersion surface tension components. The contact angle and surface energy were calculated as an average of 10 measurements and were expressed as mean ± standard deviation (SD). Both surfaces of each film (AS, GS) were analyzed.

#### 2.3.2 Raman imaging

Raman analysis was performed using a Confocal Raman Imaging system Witec alpha 300 M + equipped with a 100 × air objective (Zeiss EC Epiplan-Neofluar Dic, NA = 0.9). The measurements were conducted using an air-cooled solid-state laser operating at 488 nm. Raman images were obtained with the spatial resolution of 0.5 μm with integration time for single spectrum of 1s and a spectral resolution of 3 cm^−1^. Data analysis were performed using WITec Project Plus and OPUS software. Collected spectra were pre-processed by removing cosmic rays, smoothing and vector normalization in the whole spectral range. All Raman images were analyzed after pre-processing using cluster analysis (CA) method with the k-means algorithm and in the Manhattan-distance formulation.

#### 2.3.3 Evaluation of mechanical properties

Tensile strength (σ_M_), Young's modulus (E_t_), and elongation at maximum force (ε_M_) were determined using a universal testing machine Inspect Table Blue 5 kN with 100 N load cell (Hegewald&Peschke, Germany). The sample dimensions were 30 mm × 5 mm. The pre-load force was 0.1 N, and the test speed was 10 mm min^−1^. Mechanical properties were calculated by averaging 10 measurements and were expressed as mean ± standard deviation (SD).

#### 2.3.4 Evaluation of structural and thermal properties

Wide-angle X-ray scattering (WAXS) measurements were performed using an X-ray diffractometer X'Pert Pro (PANalytical, Netherlands) with Cu-Kα radiation in the 2θ 10°–35° range and at 5° min^−1^. The Scherrer equation was used to estimate the PCL crystallite sizes (L_hkl_) perpendicular to the (hkl) plane, from the X-ray diffraction peaks broadening as follows: L_hkl_ = Kλ/βcosθ, where K is the Scherrer shape factor (0.89), λ is the X-ray wavelength (0.15406 nm), θ is diffraction angle, and β is the pure line broadening: $\beta =\sqrt{B^{2}-b_{0}^{2}}$*,* where B is the experimentally measured full-width at half-maximum (FWHM), and b_0_ is the instrumental broadening (0.0565°).

The degree of crystallinity and melting temperature of PCL were measured with power compensation differential scanning calorimetry (DSC, PerkinElmer DSC-7, USA) during a single heating run. The degree of crystallinity (χc) was estimated using the enthalpy of melting change according to the equation: χc = ΔH_m_/(1 – x – y)ΔH^0^_m_, where ΔH_m_ and ΔH^0^_m_ were the enthalpies of melting of the sample and the fully crystalline PCL (139.5 J g−1) [29] respectively, x was the weight fraction of the BG particles, and y was the weight fraction of the PPh. Measurements were conducted in standard aluminum pans. The specimens were scanned at the 20–100 °C temperature range with a heating rate of 10 °C min^−1^, using nitrogen as a purge gas.

Fourier transform infrared (FTIR) spectra were collected using spectrometer Vertex 70V (Bruker, USA) equipped with ZnSe crystal ATR unit. Measurements were conducted in the 550–4000 cm^−1^ wavenumber range with 128 scans and resolution of 4 cm^−1^. Both surfaces of each film (AS, GS) were analyzed.

#### 2.3.5 *In vitro* apatite-forming ability test

Apatite-forming ability on the surface of the films was assessed by incubation in simulated body fluid (SBF), prepared according to Kokubo [30] at 37 °C for 3, 7, and 14 days with the sample weight to SBF volume ratio of 10^−3^ g mL^−1^ [31]. All the reagents used for SBF preparation were purchased from Avantor Performance Materials, Poland. Afterwards, the samples after washing with anhydrous ethanol (Avantor Performance Materials, Poland) and drying at room temperature were examined with SEM/EDX and ATR-FTIR methods as mentioned above. Both surfaces of each film (AS, GS) were tested. Furthermore, inductively coupled plasma atomic emission spectrometry (ICP-OES; Plasm 40, PerkinElmer, USA) was used to evaluate the changes in the concentrations of Ca, P, and Si in the SBF during incubation of the films. The measurements were performed in triplicate and expressed as mean ± standard deviation (SD).

#### 2.3.6 *In vitro* polyphenol release and antioxidant activity testing

The kinetics of PPh release from films was evaluated by incubation in phosphate-buffered saline (PBS; pH = 7.4, HyClone, USA) at 37 °C for 120 h with the sample weight to medium volume ratio of 8·10^−3^ g mL^−1^. After the predetermined time, the medium was collected and replaced with the same volume of fresh PBS. PPh concentration in PBS was determined spectrophotometrically using a microplate reader (POLARstar Omega, BMG Labtech, Germany) at 320 nm. The wavelength used to determine the concentration of PPh in PBS was selected based on the UV–Vis spectrum of extract solution in the 200–1100 nm wavelength range. The concentration of released PPh in PBS was determined based on calibration curve, while the percentage of released PPh was calculated with respect to the initial content of PPh incorporated in the materials.

The HPLC analysis of PPh in extract and media after 72-h incubation was conducted using the Prominence-i LC-2030C 3D Plus system (Shimadzu, Japan) equipped with a diode array detector (DAD). The extract was dissolved in the incubation medium to the concentrations corresponding to the polyphenol content in the films (PCL/4.5PPh, PCL-A2/4.5PPh). The separation was performed on the Luna Omega 5 μm Polar C18, 100 A, 250 × 10 mm column (Phenomenex, California, USA) at 40 °C. The mobile phase was a mixture of two eluents: A – 0,1% v/v formic acid in UHQ water and B – 0,1% v/v formic acid in methanol. The flow rate of the mobile phase was 1,2 mL/min. The analysis was carried out with the following gradient conditions: from 20% to 40% B in 10 min, 40% B for 10 min, from 40% to 50% B in 10 min, from 50% to 60% B in 5 min, 60% B for 5 min, from 60% to 70% B in 5 min, from 70% to 90% B in 5 min, 90% B for 5 min, from 90% to 20% B (the initial condition) in 1 min and 20% B for 4 min, resulting in a total run time of 60 min. The injection volume was 20 μL. All of the reagents used for HPLC analysis were purchased from Sigma-Aldrich, Germany.

Antioxidant activity of the materials was evaluated using ABTS and DPPH free radical scavenging assays and ferric reducing antioxidant power (FRAP) test [32,33]. The samples were incubated with shaking in ABTS, DPPH and FRAP working solutions for 10 min in the dark at 30 °C. The sample weight to working solution volume ratio was 10^−3^ g mL^−1^. For ABTS DPPH, and FRAP assays, the changes of absorbance at 734 nm, 515 nm, and 593 nm respectively, were measured using a spectrometer (UV-1800, RayLeigh, China). The radical scavenging capacity (RSC) of the materials was calculated according to the following equation: RSC=(A_0_–A_S_/A_0_), where A_S_ was the absorbance of the solution after sample incubation, and A_0_ was the absorbance of ABTS and DPPH working solutions. The results of the FRAP test was expressed as absorbance. Polyphenol release and antioxidant activity tests were performed in triplicate and expressed as mean ± standard deviation (SD).

#### 2.3.7 *In vitro* cell culture testing

For the cell culture, both surfaces of each sample were sterilized with UV-C light for 15 min each and washed with sterile phosphate-buffered saline (PBS, HyClone, USA). The sterile films were placed at the bottom of 48-well culture plate wells (Nunc™, Denmark) and held down by inserts made of ultrapure silica glass (Continental Trade, Poland) to prevent samples from floating. Cells were seeded on the GS surface of films [34]. The bottom surfaces of tissue culture polystyrene (TCPS) wells served as a control.

The human normal tracheal fibroblasts (Hs680.Tr, ATCC, USA) were used for material cytocompatibility evaluation. Fibroblasts were cultured in Dulbecco's Modified Eagle's Medium (DMEM, ATCC, USA) containing 10% Fetal Bovine Serum (FBS) according to the manufacturer's protocol at a density of 2·10^4^ cells/mL/well in 1 mL of culture media for 3 and 7 days. The metabolic activity of cells after 3 and 7 days of culture was evaluated using the PrestoBlue Cell Viability Reagent (Invitrogen, USA) according to the manufacturer's protocol. Fluorescence was measured at 560/590 nm (excitation/emission) using a plate reader (POLARstar Omega, BMG Labtech, Germany). The proliferation rate of cells and cytotoxicity of obtained films were assessed using the ToxiLight™ BioAssay Kit and ToxiLight™ 100% Lysis Reagent Set (Lonza, USA) according to the manufacturer's protocol. The kit was used to quantify adenylate kinase (AK) in both supernatant (representing damaged cells) and lysate (representing intact adherent cells). The results were expressed as mean ± standard deviation (SD) from 8 samples for each experimental group.

The Normal Human Osteoblasts (NHOst, Lonza, USA) were used for evaluation of the osteogenic potential of the materials. Osteoblasts were expanded in complete osteoblast growth medium OGM BulletKit (Lonza, USA) containing 10% FBS, 0.1% ascorbic acid and 0.1% GA-1000 (Gentamicin Sulfate and Amphotericin-B). NHOst cells were seeded on the films at a density of 1.5·10^4^ cells/well in 1 mL of complete osteoblast growth medium (OGM) supplemented with differentiation kit SingleQuots (Lonza, USA), containing hydrocortisone-21-hemisuccinate and β-glycerophosphate and cultured for 7, 14, and 28 days. Osteogenic potential of the materials was assessed based on activity of the alkaline phosphatase (ALP) and also on the expression of osteocalcin (OC) and osteopontin (OP). ALP activity was evaluated using 4-methylumbelliferyl phosphate reagent (4-MUP, Sigma-Aldrich, USA). Cell lysates, obtained via cyclic freezing/thawing, were incubated with equal volumes of 4-MUP solution for 1 h. Fluorescence was determined at 360/440 nm (excitation/emission) using a plate reader (POLARstar Omega, BMG Labtech, Germany). OC and OP expression was measured using ELISA kits (Human Osteocalcin ELISA Kit and Human Osteopontin ELISA Kit, ELISAGenie, UK) according to the manufacturer's protocol. The results were normalized with respect to concerning the number of cells evaluated using the ToxiLight™ BioAssay Kit and ToxiLight™ 100% Lysis Reagent Set (Lonza, USA). The results were expressed as mean ± standard deviation (SD) from 8 samples for each experimental group.

The murine macrophages (RAW 264.7, ATCC, USA) were used for evaluation of oxidative stress in the cells. Macrophages were cultured in Dulbecco's Modified Eagle's Medium (DMEM, ATCC, USA) containing 10% Fetal Bovine Serum (FBS) according to the manufacturer's protocol at a density of 5·10^4^ cells/well in 1 mL of culture media for 1 and 3 days. The intracellular reactive oxygen species (ROS) production was measured using the cell-permeable fluorogenic probe DCFH-DA (Sigma-Aldrich, USA). The cells were washed with PBS and treated with 400 μl of l0 μM DCFH-DA solution in PBS for 30 min at 37 °C in a CO_2_ incubator. Afterwards, fluorescence was measured at 485/520 nm (excitation/emission) using a plate reader (POLARstar Omega, BMG Labtech, Germany). The results were expressed as mean ± standard deviation (SD) from 8 samples for each experimental group.

#### 2.3.8 Antibacterial testing

The reference strains used in the study were *S. aureus* DSM 24167(Deutsche Sammlung von Mikroorganismen und Zellkulturen) and *P. aeruginosa* ATCC 27853 (American Type Culture Collection). The strains were incubated in 50 mL of Bacto™ Tryptic Soy Broth (TSB) (Becton Dickinson) for 18 h at 37 °C and were harvested in the mid-exponential growth phase. Bacteria were centrifuged at 13.4 rpm for 5 min to pellet the cells and washed three times with sterile saline solution (0.9% NaCl) to remove residual macromolecules and other growth medium constituents. Then the pellets were resuspended in saline solution and thoroughly vortexed. Bacterial cell suspensions were diluted to obtain cell samples containing 10^6^ CFU mL^−1^. The experiments were performed in 24-well tissue culture plates, where each sample was placed and incubated for 4 h in 1 mL of bacteria suspension. The bottom surfaces of tissue culture plate wells served as a control. After the incubation, the samples were carefully washed three times with saline solution in order to remove non-attached bacteria cells from the surface. Then the samples were placed in a fresh 24-well plate and the adhered bacteria were mechanically removed from the surface by vivid washing with saline solution. Afterwards, the obtained suspensions were diluted in the ratio 1:10, 1:100, 1:1000 in saline solution and 100 μl of each suspension was cultured on Mueller-Hinton Agar. The plates were incubated aerobically for 24 h at 37 °C. After the incubation, colonies were counted and the number of adherent bacteria was calculated in CFU cm^−2^. Within each experiment, incubations were carried out in triplicates. The results were expressed as mean ± standard deviation (SD) for each experimental group. Furthermore, the samples with the adhered bacteria were examined with SEM method as mentioned above.

### 2.4 Statistical analysis

The results were analyzed using one-way analysis of variance (ANOVA) with Duncan post hoc tests, which were performed with Statistica 13 (StatSoft®, USA) software. The results were considered statistically significant when p < 0.05.

## 3 Results and discussion

**Fig. 1:**
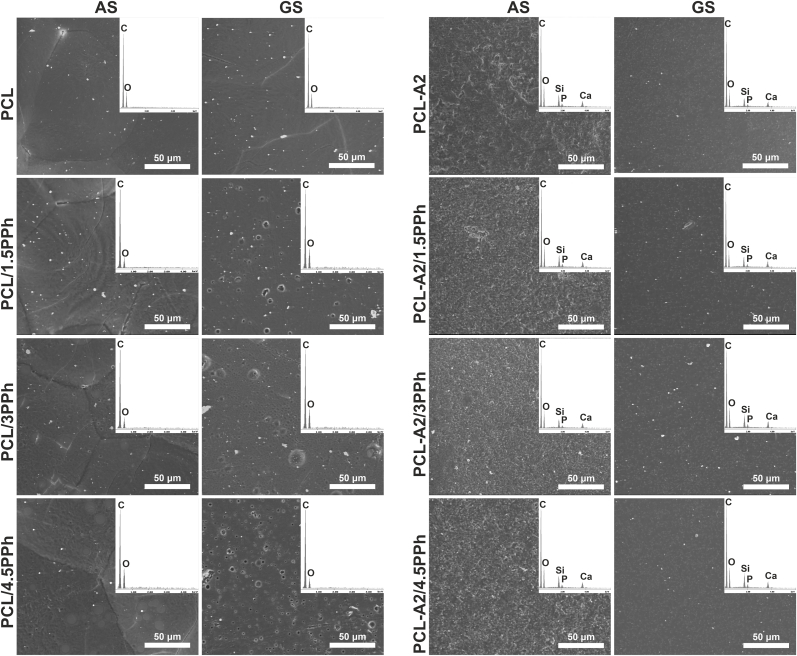
SEM images and EDX spectra (averaged for the entire analyzed surface) of the AS and GS of the films.

SEM images and EDX spectra of the AS and GS of the films are shown in Fig. 1. On the entire AS of all polymeric films, separate, spherule-like aggregations were visible. It seems that the outer boundaries of the spherulites became more distinct and the fibril-like morphology was more visible upon modification of PCL-based film with PPh. This may be due to the promotion of PCL crystallization, resulting in an increased degree of crystallinity and crystallite size of PCL upon modification with PPh (Table 2). In turn, the GS did not contain characteristic spherule-like aggregations. The differences between AS and GS of PCL-based films have been discussed in details in our previous work [34]. Moreover, the presence of PPh induced porosity of GS. The pores were homogeneously distributed on the entire surfaces of the films. The number of pores increased with increasing PPh content. The reasons for porosity development remain unclear. The AS of PCL-A2-based materials contained numerous, filamentous PCL aggregations, without typical spherule-like morphology. These surfaces showed significantly higher porosity and larger surface area compared to both surfaces of polymeric films and GS of composites. The presence of PPh in composite films seemed to increase surface are of AS. The GS of the PCL-A2-based films were uniform and nonporous. The presence of PPh did not affect GS microstructure significantly. BG particles were homogeneously distributed on GS and AS, which was confirmed by EDX elemental mapping (Fig. A1) and also Raman imaging (Fig. A2B). In the case of Raman analysis, the distribution of the BG particles was based on integration of the most intense band characteristic of BG located at 958 cm^−1^ (Fig. A2A). Furthermore, using the cluster analysis, two classes were generated (blue and red). The averaged spectra extracted from these clusters revealed bands characteristic of PCL and BG. However, the bands originating from BG (especially bands at 958 cm^−1^ and 1086 cm^−1^) showed higher intensity in red spectra, corresponding to clusters marked in red on the CA images. This indicates areas with more exposed BG particles on the surfaces and/or higher concentration of them. The presence of bands characteristic of BG in both blue and red clusters and uniform distribution of the red clusters in the analyzed regions suggested even distribution of BG particles in PCL matrix for both AS and GS.

**Fig. 2:**
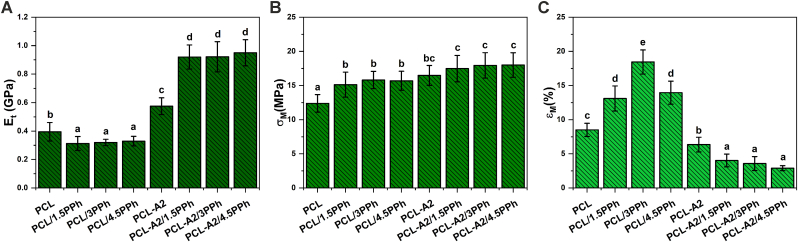
Young's modulus (A), tensile strength (B), and elongation at maximum force (C) of the films. Statistically significant differences (p < 0.05) are indicated by subsequent lower Latin letters. Different letters indicate statistically significant differences.

The results of Young's modulus (E_t_), tensile strength (σ_M_), and elongation at maximum force (ε_M_) analyses of the films are presented in Fig. 2. The values of Young's modulus of polymeric materials decreased significantly upon modification with PPh, whereas, the changes did not depend on PPh concentration. Composite materials containing BG particles showed significantly improved E_t._ Interestingly, a combination of BG particles and PPh allowed obtaining nearly 3-fold higher values of E_t_ compared to PCL/PPh materials. The introduction of PPh into both polymeric and composite materials resulted in increased σ_M_. Nevertheless, only differences noted for PCL/PPh films were statistically significant. Neither E_t_ nor σ_M_ depended on PPh concentration. In contrast, ε_M_ of the polymeric films increased almost linearly with increasing concentration of PPh up to 3%, while the composite with the highest concentration of PPh (PCL-A2/4.5PPh) showed ε_M_ similar to the value measured for PCL-A2/1.5PPh. In the case of PCL-A2-based composites, the presence of PPh significantly decreased elongation, and the values did not depend on PPh concentration. These results correlate with the values of E_t_ for composite films.

As polymeric PCL/PPh films showed significantly reduced E_t_ and increased ε_M_ compared to PCL material, polyphenols can be considered as plasticizers for PCL [35]. Moreover, the shift in melting temperature to lower values along with increasing PPh content can confirm that PCL chains interact with polyphenol molecules (Table 2) [36]. Therefore, PPh possibly reduce the intermolecular forces between polymer chains, and thus increase mobility of chains. This leads to the promotion of PCL crystallization, resulting in an increased degree of crystallinity and crystallite size of PCL upon modification with PPh (Table 2) [37]. As previously shown, plasticizing effect of semicrystalline polymers is accompanied by decrease in melting temperature and increase in degree of crystallinity [36,38]. PCL contains carbonyl groups which are hydrogen bond acceptors, while PPh because of numerous hydroxyl groups represent good hydrogen-bonding donor. Thus hydrogen bonding between these moieties can easily form [39], what can be confirmed by FTIR analysis (Fig. A3). The absorption spectrum of PPh showed a broad band at 3265 cm^−1^, which can be assigned to the hydrogen-bonded hydroxyl groups. For spectra of the PCL/PPh films, that band shifted to higher frequency, indicating a switch from the strong intramolecular hydroxyl–hydroxyl interactions to the weak intermolecular hydroxyl–carbonyl bonds. Furthermore, the successful introduction of PPh can be proved by the appearance of a new band at 1600 cm^−1^ attributed to the aromatic ring skeletal vibrations of PPh [22,40]. Jimenez et al. reported that mixing acrylic copolymer latex-containing pyrrolidone groups, as a hydrogen bond acceptors, with tannic acid, as a hydrogen bond donating polyphenol, has led to H-bond interactions, establishing materials with improved mechanical properties, i.a. tensile strength [39]. Hydrogen bonding was also confirmed by Kuo et al. [40] in polymer blends between carbonyl groups of PCL and hydroxyl groups of phenolic, poly(vinylphenol), and phenoxy.

**Fig. 3:**
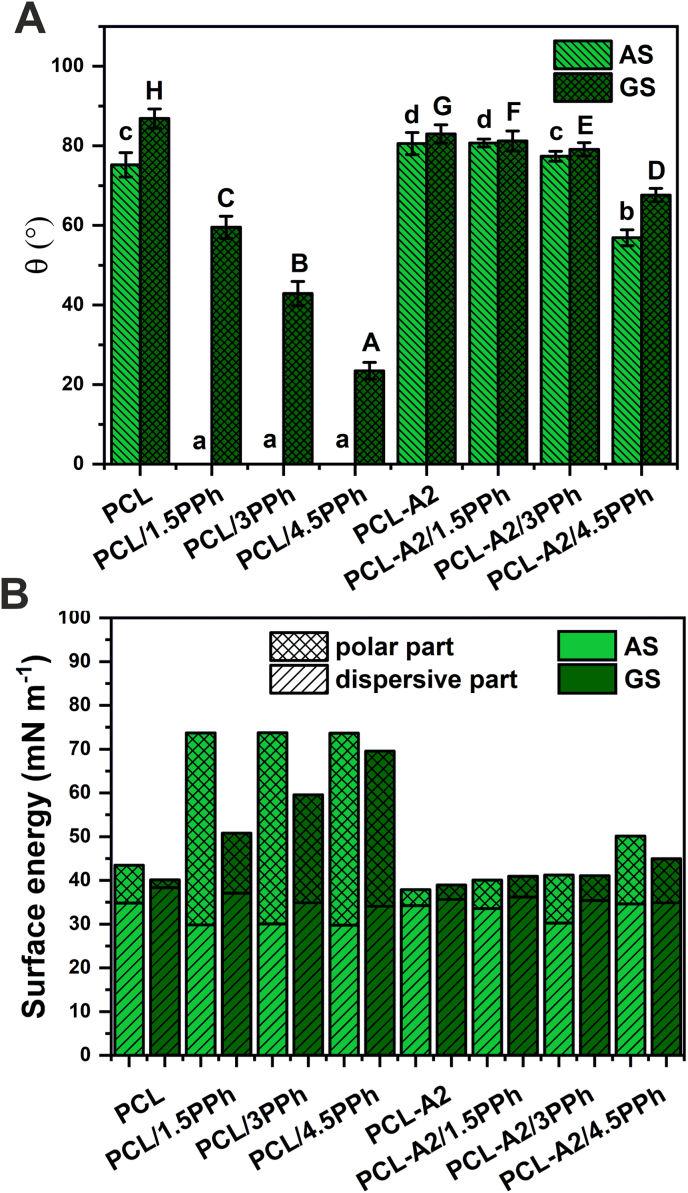
Static water contact angle (A) and total surface energy (B) with its dispersive (σ_D_), and polar (σ_P_) components of both surfaces (AS and GS) of the films. Statistically significant differences (p < 0.05) for AS and GS are indicated by subsequent lower and upper Latin letters, respectively. Different letters indicate statistically significant differences.

On the other hand, we have hypothesized that polyphenols can act as bifunctional coupling agents as they provide linkages between the fillers and the polymer matrix. That can be supported by the significantly improved mechanical characteristics, especially Young's modulus, of composite films containing PPh. It should be pointed out that BG particles had been mixed with plant extract and stirred for 24 h before having added PCL to allow PPh binding to the fillers. As was shown by Cazzola et al. [[41], [42], [43]] as well as Ferraris and Zhang et al. [[44], [45], [46]] polyphenols can be easily coupled with the surface of bioactive glasses and glass-ceramic by simple immersion and incubation method. These authors functionalized melt-derived BG with polyphenols after surface activation to expose –OH groups. In this work, sol-gel derived BG with high surface area and rich in surface silanol groups (Si–OH) were used. Therefore, highly effective binding capacity by the formation of hydrogen bonds between numerous hydroxyl groups of BG and PPh was expected. To sum up, polyphenols can be considered as green plasticizers in polymeric materials and as coupling agents in composites for biomedical applications to modulate mechanical features. In addition, PPh can provide and/or modify other properties, as described below.

The results of analyses of static water contact angle (θ) and surface energy (SE) with its dispersive (σ_D_) and polar (σ_P_) components of both surfaces (AS and GS) of the films are shown in Fig. 3. AS of unmodified PCL film was more hydrophilic, compared to its GS. The modification of that film with PPh significantly improved the wettability of both surfaces, but in a different manner. Upon modification with PPh, AS became completely wettable, even for the lowest concentration of PPh, while the values of θ observed for GS decreased almost linearly with increasing concentration of PPh. The presence of BG particles in the PCL matrix significantly modified surface wettability – contact angle of AS increased, while its value for GS decreased. PPh in PCL-A2-based composites improved the wettability of both surfaces (the only exception was AS of PCL-A2/1.5PPh film, which did not show significant differences compared to PCL-A2 material), however the changes were not as pronounced as those observed for PCL/PPh films. The highest concentration of PPh (4.5%) in the PCL-A2-based film resulted in the highest changes in contact angle values of both surfaces. Just like with polymeric films, more pronounced reduction of θ was observed for AS. It was found that the presence of PPh in PCL films significantly increased the total surface energy of both of the surfaces (Fig. 3B). That resulted mainly from the increase in the polar component, what correlates with a significant improvement in wettability. The surface energy, as well as both σ_D_ and σ_P_ components of AS of PCL/PPh materials, were on a similar level, regardless of PPh concentration. Simultaneously, σ_D_ of GS of PCL/PPh films did not change, while σ_P_ increased with increasing PPh content. The modification of the PCL matrix with BG particles did not influence the total surface energy of GS, while SE of AS was slightly lower what was accompanied by a decrease in σ_P_. In the case of PCL-A2/PPh composites, increasing concentration of PPh resulted in increased σ_P_ of both surfaces with tendency to increased SE, whereas, the effect was more pronounced for AS. The presence of the highest concentration of PPh (4.5%) resulted in the highest changes in SE of both surfaces.

After modification with PPh, the polymeric films revealed significantly improved hydrophilicity and increased the polar part of surface energy what can be attributed to the presence of –OH groups of the PPh exposed on the surfaces of materials. A similar observation was made by Kim et al. [47], who modified electrospun PCL nanofibers with phlorotannin. The differences in wettability and surface energy between AS and GS of PCL/PPh films probably have arisen from the various concentrations of PPh on those surfaces. Polyphenols, because of their relatively low molecular weight, show high mobility in solutions. During the solvent evaporation from the polymer solution, migration of solvent molecules to the AS surface of the film occurred, spurring at once the movement of PPh molecules. That resulted in a higher concentration of PPh on AS, so that even the lowest amount of PPh in the materials made this surface completely wettable. That can be confirmed by FTIR analysis, as the bands at 1600 cm^−1^ and 3265 cm^−1^ showed higher intensity for AS of the PCL/PPh films (Fig. A3). In the case of composite films, only the highest concentration of PPh in materials led to lowering the values of surface energy and water contact angle. These results support the findings that BG particles effectively bind PPh, reducing their concentration on the surfaces of films.

**Fig. 4:**
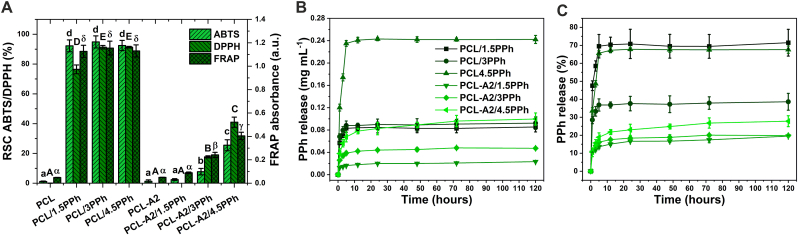
Radical scavenging capacity (RSC) against the ABTS^•+^ and DPPH^•^ radicals, as well as ferric reducing antioxidant potential (FRAP) of the films (A). Statistically significant differences (p < 0.05) for ABTS, DPPH, and FRAP assays are indicated by subsequent lower, upper Latin letters and Greek letters, respectively. Different letters indicate statistically significant differences. The release profiles of polyphenols presented as PPh concentration in release medium (B) and as percentage of released PPh in reference to the initial content in the films (C).

The radical scavenging capacity (RSC) against the ABTS^•+^ and DPPH^•^ radicals, as well as the ferric reducing antioxidant potential (FRAP) of the films, are shown in Fig. 4A. The antioxidant potential of the materials can be ascribed to the presence of PPh. PCL/PPh films showed very high RSC and reducing potential. Nevertheless, their antioxidant capacity did not depend on polyphenol concentration. Composites loaded with PPh exhibited significantly lower antioxidant potential compared to PCL/PPh films. The values measured for PCL-A2-based film with the lowest dose of PPh (1.5%) were on a similar level to those observed for unloaded PCL-A2 and PCL materials. In the case of PCL-A2/3PPh and PCL-A2/4.5PPh composites, RSC and reducing potential increased with increasing PPh concentration. However, their values for composite with the highest concentration of PPh were more than 2- to 3-fold lower when compared to PCL/PPh materials, depending on the assay used.

The release profiles of polyphenols from the materials are presented in Fig. 4B and C as a PPh concentration in release medium (mg ml^−1^) and as a percentage of released PPh in reference to the initial content of PPh in the films (%), repetitively. All of the tested materials exhibited the initial burst release of PPh in the first few hours, but the presence of BG particles reduced that effect. In the case of polymeric materials, the maximal concentrations of PPh in the medium were achieved just after 5 h, while for composite materials, the release was delayed and reached maximum values after 72 h. When considering the PPh concentration in the medium (Fig. 4B), the release rate of active compounds from PCL-A2/PPh films increased with their increasing concentration in materials. Moreover, the percentage of PPh released increased in a similar manner (Fig. 4C). PCL film loaded with the lowest concentration of PPh (PCL/1.5PPh) released the lowest dose of the active compounds (0.085 mg mL^−1^) among the films from the polymeric group, and it represented 71% of the initial content of PPh in that material. It was the highest percentage of PPh released, and similar to one released from the film with the highest concentration of PPh (PCL/4.5PPh).

HPLC analysis showed that the extract was rich in phenolic acids, phenolic diterpenes, and flavonoids (Table 1). The main polyphenols identified in the extract were rosmarinic acid, epicatechin, carnosol, and carnosic acid. The analysis of medium after 72-h incubation confirmed that polymeric film (PCL/4.5PPh) released a significantly higher amount of PPh in comparison with composite material (PCL-A2/4.5PPh). It was noted that individual polyphenols exhibited various degrees of release from the materials. What is more, the significant differences in the PPh release rate were noted between polymeric and composite films.

**Table 1:** HPLC analysis of the extract and media after 72 h of material incubation.

Polyphenol	Extract	PCL/4.5PPh film		PCL-A2/4.5PPh film	
	Concentration (mg dm^−3^)	Concentration (mg dm^−3^)	% of release	Concentration (mg dm^−3^)	% of release
Phenolic acids					
Gallic acid	0.09 ± 0.00	0.00 ± 0.00	0	0 ± 0.00	0
Chlorogenic acid	0.75 ± 0.18	0.56 ± 0.02	74	0.31 ± 0.01	41
Caffeic acid	1.85 ± 0.00	1.86 ± 0.00	100	0.96 ± 0.00	52
Vanillic acid	0.15 ± 0.00	0.11 ± 0.00	72	0.12 ± 0.00	83
Syringic acid	2.31 ± 0.01	2.01 ± 0.07	87	0.74 ± 0.04	32
*p*-Coumaric acid	0.15 ± 0.01	0.15 ± 0.01	100	0.14 ± 0.01	95
Ferulic acid	1.41 ± 0.00	0.66 ± 0.00	47	0.77 ± 0.00	55
Sinapinic acid	0.23 ± 0.02	0.19 ± 0.01	85	0.21 ± 0.00	94
Rosmarinic acid	174.13 ± 0.09	160.26 ± 0.12	92	10.79 ± 0.01	6
4-Hydroxybenzoic acid	1.19 ± 0.02	0.39 ± 0.03	33	0.93 ± 0.00	79
Flavonoids					
Naringin	4.32 ± 0.18	3.72 ± 0.00	86	0.48 ± 0.01	11
Rutin	1.55 ± 0.09	0.59 ± 0.50	38	1.24 ± 0.01	81
Hesperidin	0.08 ± 0.00	0.08 ± 0.69	97	0.07 ± 0.01	93
Myricetin	2.20 ± 0.14	1.37 ± 0.02	62	0.54 ± 0.00	24
Quercetin	0.89 ± 0.00	0.8 ± 0.04	90	0.63 ± 0.00	70
Luteolin	0.28 ± 0.01	0.27 ± 0.03	97	0.28 ± 0.00	99
Kaempferol	0.34 ± 0.00	0.28 ± 0.00	81	0.23 ± 0.00	68
Apigenin	0.26 ± 0.01	0.19 ± 0.00	72	0.11 ± 0.00	41
Isorhamnetin	0.45 ± 0.01	0.32 ± 0.00	71	0.33 ± 0.01	73
Hispidulin	0.47 ± 0.01	0.37 ± 0.01	78	0.42 ± 0.00	89
Epicatechin	15.45 ± 0.08	13.78 ± 0.24	89	0.24 ± 0.03	2
Catechin	1.49 ± 0.01	1.32 ± 0.05	88	1.49 ± 0.02	100
Acacetin	0.16 ± 0.00	0.15 ± 0.00	98	0.37 ± 0.37	90
Phenolic diterpenes					
Carnosol	47.35 ± 0.03	46.83 ± 0.11	99	0.00 ± 0.01	0
Carnosic acid	7.10 ± 0.10	4.73 ± 0.06	67	4.99 ± 0.00	70

**Table 2:** Melting temperature (T_m_), degree of crystallinity (χ_c_), and crystallite sizes (L_110_ and L_200_) of the PCL matrix of the films.

Material	DSC			WAXS	
	T_m_ (°C)	X_C_ (%)	L_110_ (nm)		L_200_ (nm)
PCL	65.2	29.3	8.9		10.2
PCL/1.5PPh	65.2	44.2	11.7		12.8
PCL/3.0PPh	65.0	44.0	20.9		18.5
PCL/4.5PPh	64.4	41.2	24.3		20.8
PCL-A2	65.0	45.0	19.5		15.5
PCL-A2/1.5PPh	64.6	42.8	15.5		15.5
PCL-A2/3.0PPh	64.2	39.7	14.4		12.8
PCL-A2/4.5PPh	64.1	34.2	12.7		13.9

The decreased antioxidant activity and the reduced initial burst release effect observed for composite films were associated with the above-mentioned PPh binding capacity of BG particles. Similar observations were made by Shao et al. [48], who used multi-walled carbon nanotubes as a modifier of the PCL matrix. Furthermore, the differences in the release degree between individual polyphenols, as well as between polymeric and composite films could have resulted from different size and structure of polyphenol molecules in plant extract, as well as from various number and arrangement of the phenolic hydroxyl groups, involved in PPh-PCL and PPh-BG bonding. In the case of PCL-A2/PPh composites, the results showed a correlation between the radical scavenging capacity/ferric reducing antioxidant potential and release profiles of PPh. However, it should be highlighted that not only PPh released from the materials were responsible for their antioxidant activity but also those present on the film surfaces. The above-mentioned correlation was not observed for PCL/PPh materials. This is probably because of markedly higher concentration of PPh on the surfaces of PCL/PPh films, providing maximal antioxidant effect even with the lowest concentration of PPh (PCL/1.5PPh). Importantly, the antioxidant activity of PPh depends mainly on the total number of phenolic hydroxyl groups able to interact with ROS by donating hydrogens [49]. Therefore, phenolic hydroxyl groups involved in hydrogen bonding with –OH groups of BG and carbonyl groups of PCL were not available to scavenge radicals and to reduce ferric ions. In summary, the *in vitro* release kinetics and antioxidant capacity can be controlled by using BG particles with high PPh binding capacity as polymer matrix fillers.

The phenolic diterpenes, carnosol and carnosic acid, as well as the phenolic acid – rosmarinic acid, are the main antioxidant components that are largely responsible for biological activities of sage [50]. Rosmarinic acid was found to increase the alkaline phosphatase activity and to induce the mineralization in osteoblasts, as well as to inhibit the osteoclast formation *in vitro* [51]. It was shown that carnosol and carnosic acid exhibited strong antibacterial activity [52,53]. Other studies revealed that plant extracts rich in carnosic acid and rosmarinic acid exhibit anti-inflammatory properties [54]. Therefore, sustained release of polyphenolic compounds and controlled antioxidant activity of biomaterials is essential for developing bone implants that actively support bone regeneration.

**Fig. 5:**
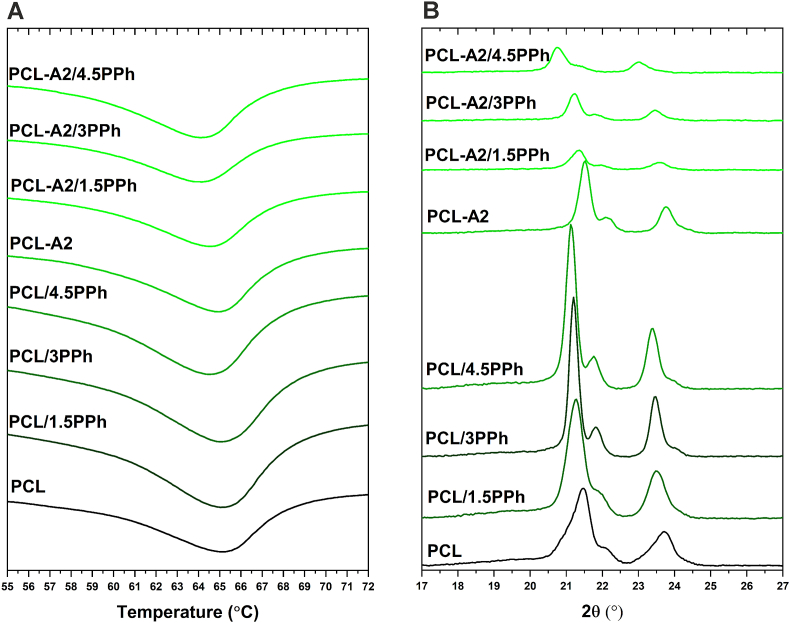
DSC thermograms (A) and WAXS patterns (B) of the films.

DSC thermograms and WAXS patterns of the films are presented in Fig. 5. Moreover, the results of the melting temperature (T_m_), degree of crystallinity (χ_c_), and crystallite sizes (L_110_ and L_200_) of the PCL matrix of the films, calculated based on the DSC and WAXS analyses, respectively, are shown in Table 2. The presence of BG particles in the PCL matrix, without PPh, did not change T_m_, but both crystallinity and crystallite sizes increased significantly. Melting temperature of PCL in both polymeric and composite materials tended to decrease upon modification with PPh. In the group of PCL-based materials, the presence of PPh resulted in increased χ_c_, as well as L_110_ and L_200_. Furthermore, in the WAXS patterns of these films, the intensity of reflexes increased and their full-width at half-maximum decreased, confirming increasing crystallinity of PCL. In contrast, crystallinity and crystallite sizes of the PCL matrix in composite films decreased with increasing PPh concentration.

**Fig. 6:**
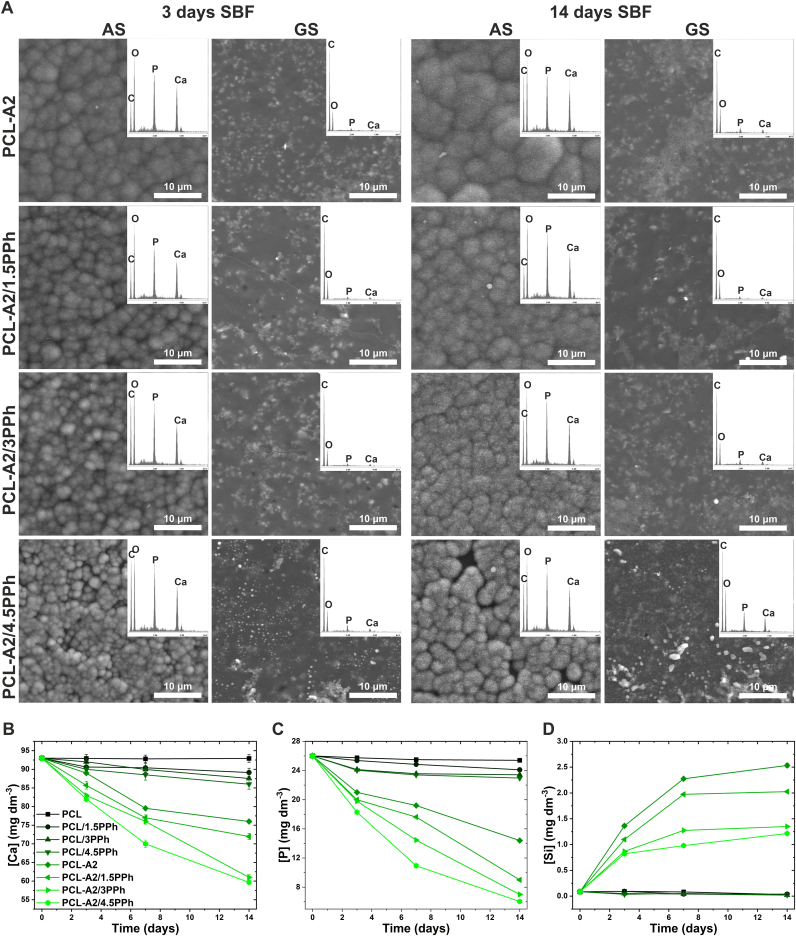
SEM images and EDX spectra (averaged for the entire analyzed surface) of the AS and GS of the films after 3- and 14-day incubation in SBF (A). The changes of Ca (B), P (C), and Si (D) concentrations in SBF during incubation of the films.

The films were evaluated in terms of apatite-forming ability during 14-day incubation in SBF. SEM images and EDX spectra of GS and AS of the films after incubation are presented in Fig. 6A. Both surfaces of PCL and PCL/PPh films did not show any chemical and morphological changes even after 14 days of incubation (data not shown). After 3- and 14-day incubation, GS of all composite films were covered with a uniform layer containing small amounts of Ca and P. Layer did not show morphology typical of hydroxyapatite precipitated from SBF. After 3 days, spherical cauliflower-like crystals rich in Ca and P, characteristic of carbonated hydroxyapatite (HCA), appeared on the AS of PCL-A2 and PCL-A2/PPh composites. The size of depositions decreased with increasing PPh concentration and they become bigger after a longer incubation period. A lack of Si from BG indicated that the layers were thick and homogeneously covered the surfaces of films [31]. The differences in the formation of CaP layer between AS and GS could have occurred because of various surface microstructures, especially larger surface area of AS compared to GS of composites [55].

**Fig. 7:**
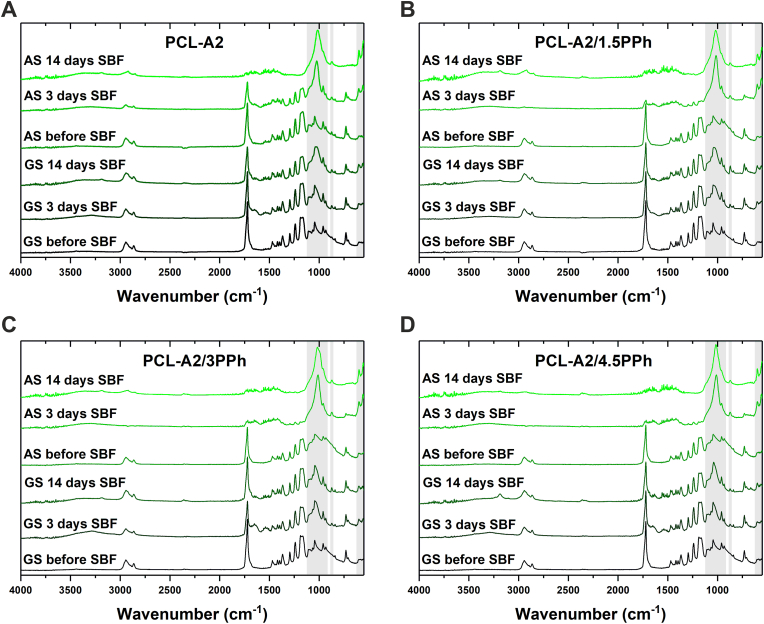
ATR-FTIR spectra of AS and GS of the PCL-A2 (A), PCL-A2/1.5PPh (B), PCL-A2/3PPh (C), PCL-A2/4.5PPh (D) composite films before as well as after 3- and 14-day incubation in SBF.

The formation of calcium phosphate (CaP) layer on both surfaces of the films, during incubation in SBF, was evaluated using ATR-FTIR spectroscopy (Fig. 7A–D). The results confirmed no significant changes during the incubation of PCL and PCL/PPh films (data not shown). In the case of all composite materials, new bands in the 560–602 cm^−1^ and 910–1180 cm^−1^ ranges, assigned to O–P–O bending mode and P–O stretching mode, respectively, appeared just after 3-day immersion in SBF. Furthermore, the low intensity band at 875 cm^−1^ could arise from P–OH stretching mode of HPO_4_^2−^ ions and/or out-of-plane bending mode of CO_3_^2−^ ions. These new bands are typical of nanocrystalline nonstoichiometric apatite [56]. The gradual increase in the intensity of bands characteristic of apatite and the reduction in the intensity of bands arising from PCL indicated increasing thickness of the layer with increasing immersion period. However, a significantly faster reduction in the intensity of PCL bands was observed for AS compared to GS, confirming accelerated and more effective apatite layer formation on those surfaces.

The results of the ICP-OES analysis of SBF during film incubation are shown in Fig. 6B–D. No significant changes in Ca, P, and Si concentrations in SBF were noted for PCL film over immersion time. In the case of PCL/PPh and PCL-A2/PPh materials, Ca and P concentrations gradually decreased, but those changes were significantly higher for composite materials, confirming apatite layer formation on their surfaces. It can be observed that the rate of Ca and P reduction increased with increasing PPh content in both polymeric and composite materials. The highest depletion was observed for the PCL-A2/4.5PPh film. Those findings coincided with the release profile of Si from composites. The release rate of Si to SBF from composite films with higher PPh concentrations was significantly reduced, compared to materials without PPh or with lower PPh content. This was due to accelerated CaP layer development on the surface of composites, which slowed down the release of Si. Furthermore, after 7 days of incubation, when the layer was well developed, the Si release rate from the films was significantly reduced.

The results indicated that the presence of PPh accelerated the formation of an apatite layer on the surface of composites. Similar observations were made by Cazolla et al. who functionalized the surface of melt-derived BG with gallic acid and natural polyphenols extracted from red grape skins and green tea leaves [43]. That effect could have resulted from the presence of additional phenolic hydroxyl groups on the surface of materials and their ability to interact with calcium ions from physiological solution [57,58]. Therefore, they could have acted as the nucleation centers for the biomimetic apatite. That might be supported by smaller size of depositions observed for composite materials with increasing PPh concentration, especially after 3 days of incubation. Furthermore, in contrast to the neat PCL film, the surfaces of PCL/PPh materials showed the ability to adsorb calcium and phosphate ions from SBF, as was found in ICP analysis. These processes can also be fostered by improved wettability, increased surface energy, especially its polar component [59]. Another factor responsible for the accelerated formation of the CaP layer on the surfaces of composite films may be increased surface area of AS, observed for materials modified with PPh. According to the literature data, surface area and topography have been shown to influence hydroxyapatite nucleation to a great extent [33,60].

**Fig. 8:**
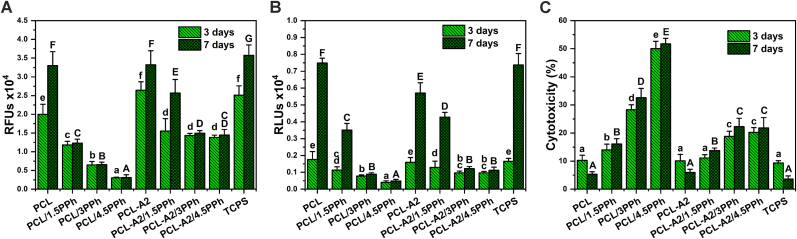
The response of Hs680.Tr fibroblasts: metabolic activity (A), adenylate kinase (AK) level in the lysate corresponding to the number of intact adherent cells (B), AK level in the supernatant related to AK level in the lysate representing material cytotoxicity (C). Statistically significant differences (p < 0.05) between films and TCPS after 3- and 7-day cell culture periods are indicated by lower and upper cases, respectively. Different letters indicate statistically significant differences.

As shown in Fig. 8A and B, the presence of PPh in PCL- and PCL-A2-based films significantly reduced metabolic activity and number of the fibroblasts after both culture periods. In the case of polymeric materials, the reduction was proportional to the increasing concentration of PPh. Although a significant decrease was noted for the PCL-A2/1.5PPh material, it was not as big as for the PCL/1.5PPh film. For composite films enriched with 3 and 4.5% of PPh, metabolic activity and number of the cells were on a similar level. What is more, a significant increase in the number of the cells between 3 and 7 days of culture on films with the lowest PPh concentration (PCL/1.5PPh and PCL-A2/1.5PPh) was observed, indicating those materials supported cell proliferation. Moreover, the results indicate relatively high metabolic activity of the cells seeded on composites containing PPh, despite the reduced number of cells. The results of metabolic activity and the number of cells coincided with the cytotoxicity of those materials (Fig. 8C). Cytotoxicity of the polymeric materials increased with increasing PPh concentration. In the case of composites, the highest values were recorded both for PCL-A2/3PPh and PCL-A2/4.5PPh. What is important, cytotoxicity of materials containing PPh did not increase significantly over time. That might confirm that the maximum release of PPh occurred after 72 h for all materials. Cytotoxicity of the polymeric and composite films with the lowest concentration of PPh (PCL/1.5PPh and PCL-A2/1.5PPh) did not exceed 20% and 15%, respectively.

Higher metabolic activity and a higher number of the cells, as well as lower cytotoxicity observed for composite materials were in agreement with reduced PPh release from these materials and also with lower concentrations of PPh on the surfaces of composite films, resulting from PPh binding capacity of BG particles.

Zadeh et al. [61] showed that the incorporation of polyphenols extracted from date palm fruit into PLA electrospun scaffolds improved proliferation and viability of NIH/3T3 fibroblasts. According to the authors, these results were attributed to increased hydrophilicity of the scaffolds. In turn, Pitz et al. reported that extract of jaboticaba fruit peels, rich in polyphenolic compounds, can support L929 fibroblast viability and proliferation up to dose of 100 μg mL^−1^, however higher concentration of polyphenols was cytotoxic [62]. Considering the results of *in vitro* cell tests, it should be taken into account that the dose of PPh which is cytotoxic in static cell culture conditions would be beneficial for cell functions in dynamic conditions and/or *in vivo*.

For further *in vitro* cell tests using osteoblasts and macrophages, materials with the lowest dose of PPh (PCL/1.5PPh and PCL-A2/1.5PPh) were chosen.

**Fig. 9:**
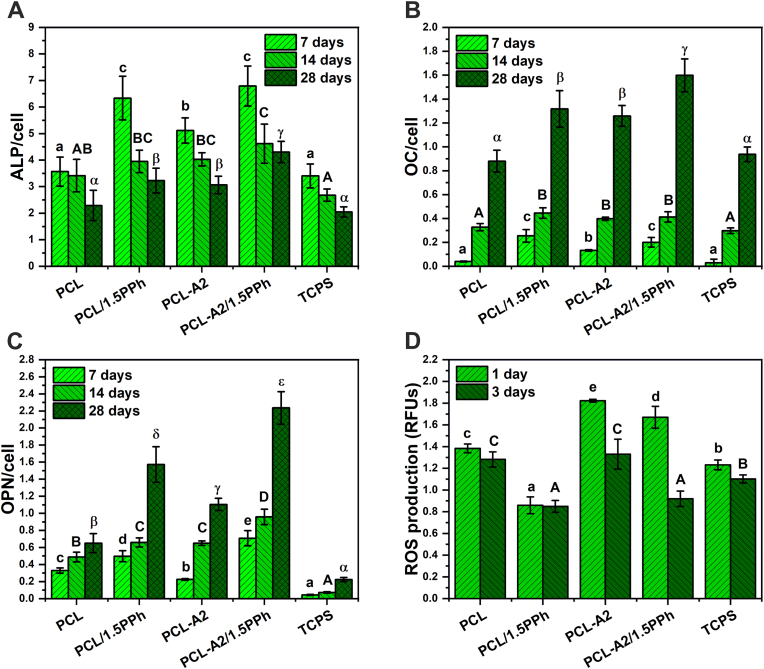
The response of NHOst: ALP activity (A), OC (B), and OP (C) expression. The response of RAW 264.7 macrophages: intracellular ROS production (D). Statistically significant differences (p < 0.05) between films and TCPS after 7-, 14-, and 28-day cell culture periods are indicated by subsequent lower, upper Latin letters and Greek letters, respectively. Different letters indicate statistically significant differences.

After 7 days of culture, osteoblasts seeded on materials containing PPh exhibited significantly higher ALP activity compared to cells in contact with PCL and PCL-A2 films, as well as TCPS (Fig. 9A). Osteoblasts cultured on PCL-A2 film also showed significantly higher activity in comparison with osteoblasts cultured in contact with PCL and TCPS. As the ALP is an early marker of osteoblast differentiation, after longer cell culture periods (14 and 28 days), ALP activity was decreasing, but the value recorded for PCL-A2/1.5PPh composite after 28-day culture was still the highest compared to the other materials and TCPS. The expression of late stage markers of osteoblast differentiation - OC and OP was increasing over cell culture period and reached the highest value on a day 28 (Fig. 9B and C). The presence of both PPh and BG particles in materials (PCL/1.5PPh, PCL-A2, and PCL-A2/1.5PPh) resulted in significantly higher OC and OP expression (the only exception was the level of OP expressed by osteoblasts in contact with PCL-A2 film after 7-days culture, which was lower compared to the value recorded for PCL material). The highest OC and OP expression was observed for PCL-A2/1.5PPh composite after 28-day culture.

On the one hand, many *in vitro* studies indicated that bioactive glasses significantly improved osteoblastic cell differentiation. It was shown that BG enhanced ALP activity, extracellular matrix (ECM) mineralization, as well as the gene and ECM protein expressions [[63], [64], [65]]. On the other hand, polyphenolic compounds, for example PPh extracted from dried plums, enhanced *in vitro* osteoblast alkaline phosphatase activity, extracellular matrix mineralization, and collagen I cross-linking [66]. Dai et al. [67] found that resveratrol directly stimulated cell proliferation, osteoblastic differentiation, and osteogenic gene expressions in human bone marrow-derived mesenchymal stem cells. In the present study, the synergistic effect of combining bioactive glass and polyphenolic compounds in composite materials on osteoblast differentiation was demonstrated.

The results indicated that materials without PPh (PCL, PCL-A2) stimulated intracellular ROS production. After both culture periods, ROS level decreased significantly in macrophage cells cultured on materials containing PPh (PCL/1.5PPh and PCL-A2/1.5PPh) compared to cells cultured in contact with PCL and PCL-A2 films (Fig. 9D). After 3-day of culture, the values recorded for both of the PPh-enriched materials exhibited similar level, although PCL-A2 composite without PPh induced significantly higher ROS level in the cells. Furthermore, after a longer cell culture period, macrophages seeded on PCL/1.5PPh and PCL-A2/1.5PPh showed a lower level of ROS when compared to TCPS.

All of the implanted biomaterials cause the foreign body response, including inflammation and generation of ROS. Acute inflammatory response drives tissue regeneration and angiogenesis, while excessive inflammation and chronically high levels of ROS, leading to oxidative stress, have a negative impact on the tissue healing processes and may result in implant rejection and a number of other pathological conditions [68,69]. For instance, oxidative stress activates the differentiation of pre-osteoclasts in osteoclasts, as well as induces the apoptosis of osteoblasts, inhibiting their activity and differentiation. This can delay the bone regeneration process or even lead to tissue resorption around the implant [70,71]. The production of ROS in macrophages is one of the most important characteristics in the inflammatory response [72]. It was shown that pomegranate peel polyphenols [72], as well as rosmarinic acid [73] and carnosic acid [74] inhibited the formation of ROS in RAW264.7 macrophages, indicating strong anti-inflammatory properties.

To evaluate the antibacterial activity of the investigated samples, the quantitative method of multiple dilutions was used. The summary of the experiments is presented in Fig. 9A.

**Fig. 10:**
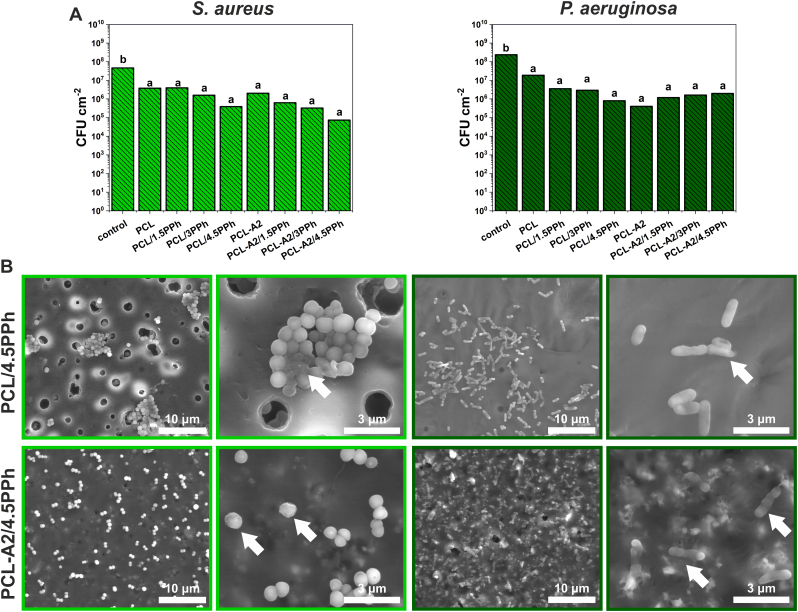
The survival rate of *S. aureus* and *P. aeruginosa* incubated with the films (A). Statistically significant differences (p < 0.05) between films and control are indicated by subsequent lower Latin letters. Different letters indicate statistically significant differences. SEM images of *S. aureus* and *P. aeruginosa* on PCL/4.5PPh and PCL-A2/4.5PPh films (B).

The strains used in the study are representative for two types of bacteria: Gram-positive (*S. aureus*) and Gram-negative (*P. aeruginosa*) which significantly differ in shape (cocci, rod) and cell wall thickness. As presented in Fig. 10A, the survival rate of both of the strains differed between investigated strains to the control sample (TCPS) with the adhesion rate of 4.7 × 10^7^ CFU/cm^2^ for *S. aureus* and 2.4 × 10^8^ CFU/cm^2^ for *P. aeruginosa*. The best antibacterial results were obtained for the PCL/4.5PPh and PCL-A2/4.5PPh films, with a survival rate of *S. aureus* was 3.9 × 10^5^ CFU/cm^2^ and 7.4 × 10^4^ CFU/cm^2^, respectively. For the rest of the materials, no significant differences were determined (values in the range 6.3 × 10^5^–4.0 × 10^6^ CFU/cm^2^), but the survival rates for those samples were one order of magnitude lower when compared to the control sample. The best results in terms of bactericidal activity for *P. aeruginosa* were obtained for PCL/4.5PPh and PCL-A2 materials with a survival rate of 8 × 10^5^ CFU/cm^2^ and 4 × 10^5^ CFU/cm^2^, respectively. Similarly, as for *S. aureus*, the rest of the samples did not differ significantly between each other (values in 1.2 × 10^6^–1.9 × 10^7^ CFU/cm^2^ range).

To gain better insight into to the morphology of microorganisms on the investigated films and to verify if the obtained quantitative results were associated with the repelling properties of the materials and their biocidal activity, SEM observations were performed.

As illustrated in Fig. 10B, the *S. aureus* bacteria attached to the investigated surfaces in a significantly various manner. At the surface of PCL/4.5PPh film, the cells tend to aggregate and form clumps without biofilm formation. Some of the cells were visibly damaged (white arrow), especially those inside the bacterial aggregates. In contrast, the *S. aureus* on sample PCL-A2/4.5PPh surface attached as single, not agglomerated cells, homogenously dispersed over the surface. As can be seen, some of the cells were shrank, with visibly damaged cell walls (white arrow).

The *P. aeruginosa* bacteria cells, similarly to the *S. aureus*, attached to the investigated surfaces in a significantly different way. For PCL/4.5PPh film, bacteria tended to cluster, but without islands-like formations as for *S. aureus*. Some of the cells were dead mostly by cell wall disruption (white arrow). The surface of PCL-A2/4.5PPh material was homogenously covered by not-clustered bacteria, but the majority of the visible biological moieties were flat with visible cell wall damages (white arrows).

Literature data have indicated that polyphenols show broad-spectrum antimicrobial activity against Gram‐positive and Gram‐negative bacteria [75] However, the mechanisms of their action have not yet been fully recognized. Cowan [76] suggested that they result from the presence of phenolic hydroxyl groups and depend on the backbone structure of PPh molecule, as well as on the number and positions of OH groups. On the other hand, highly soluble BGs also have shown antibacterial properties, as a result of massive Ca^2+^ ion release which alters pH and osmotic pressure of the environment as well as bacterial membrane potential [[77], [78], [79]]. Therefore, bacteriostatic and antibiofilm properties of PCL-A2-based composites enriched with PPh may be related to the presence of both calcium-rich BG particles and PPh. Besides the PPh binding capacity of BG particles, also PPh-BG interactions can change the activity of the composite films, altering the availability of hydroxyl groups.

## 4 Conclusions

Polymeric and BG-modified composite films were successfully loaded with PPh extracted from sage. Polyphenolic compounds significantly modified numerous properties of the materials, and also acted as biologically active substances. On the one hand, PPh can be considered as plasticizers for PCL, on the other hand, they can act as coupling agents in composite materials, improving their mechanical performance. The presence of PPh in materials improved their hydrophilicity and apatite-forming ability, and also provided antioxidant activity. What is important, the aforementioned properties and kinetics of PPh release can be modulated by the use of different concentration of PPh and by the modification of PCL matrix with sol-gel-derived BG particles, capable of binding PPh. The films containing the lowest concentration of PPh exhibited cytocompatibility, significantly increased alkaline phosphatase activity and the expression of bone extracellular matrix proteins (osteocalcin and osteopontin) in human normal osteoblasts, while they reduced intracellular reactive oxygen species production in macrophages. Furthermore, materials loaded with PPh showed antibiofilm properties against Gram‐positive and Gram‐negative bacteria. The results suggest that obtained materials represent potential multifunctional biomaterials with a wide range of tunable physicochemical and biological properties. Moreover, the proposed combination of PCL, BG particles and polyphenols can be potentially used in production of membranes and 3D scaffolds for bone tissue engineering.

## CRediT authorship contribution statement

**Michal Dziadek:** Conceptualization, Methodology, Formal analysis, Investigation, Writing - original draft, Writing - review & editing, Visualization, Supervision, Project administration, Funding acquisition. **Kinga Dziadek:** Conceptualization, Methodology, Formal analysis, Investigation, Writing - original draft, Writing - review & editing. **Kamila Checinska:** Methodology, Investigation, Writing - original draft. **Barbara Zagrajczuk:** Methodology, Investigation, Writing - original draft. **Monika Golda-Cepa:** Methodology, Investigation, Writing - original draft. **Monika Brzychczy-Wloch:** Methodology, Writing - original draft. **Elzbieta Menaszek:** Methodology, Writing - original draft. **Aneta Kopec:** Methodology, Writing - original draft, Writing - review & editing. **Katarzyna Cholewa-Kowalska:** Conceptualization, Methodology, Writing - original draft, Writing - review & editing, Supervision, Project administration, Funding acquisition.

## Declaration of competing interest

The authors declare that they have no conflict of interest.
