# Supplementary material for: PCL and PCL/bioactive glass biomaterials as carriers for biologically active polyphenolic compounds: Comprehensive physicochemical and biological evaluation
Source: Bioact Mater. 2020 Dec 5;6(6):1811–26. doi: 10.1016/j.bioactmat.2020.11.025 (PMC8484899; doi:10.1016/j.bioactmat.2020.11.025)
Supplement: Multimedia component 1 [file mmc1.docx]

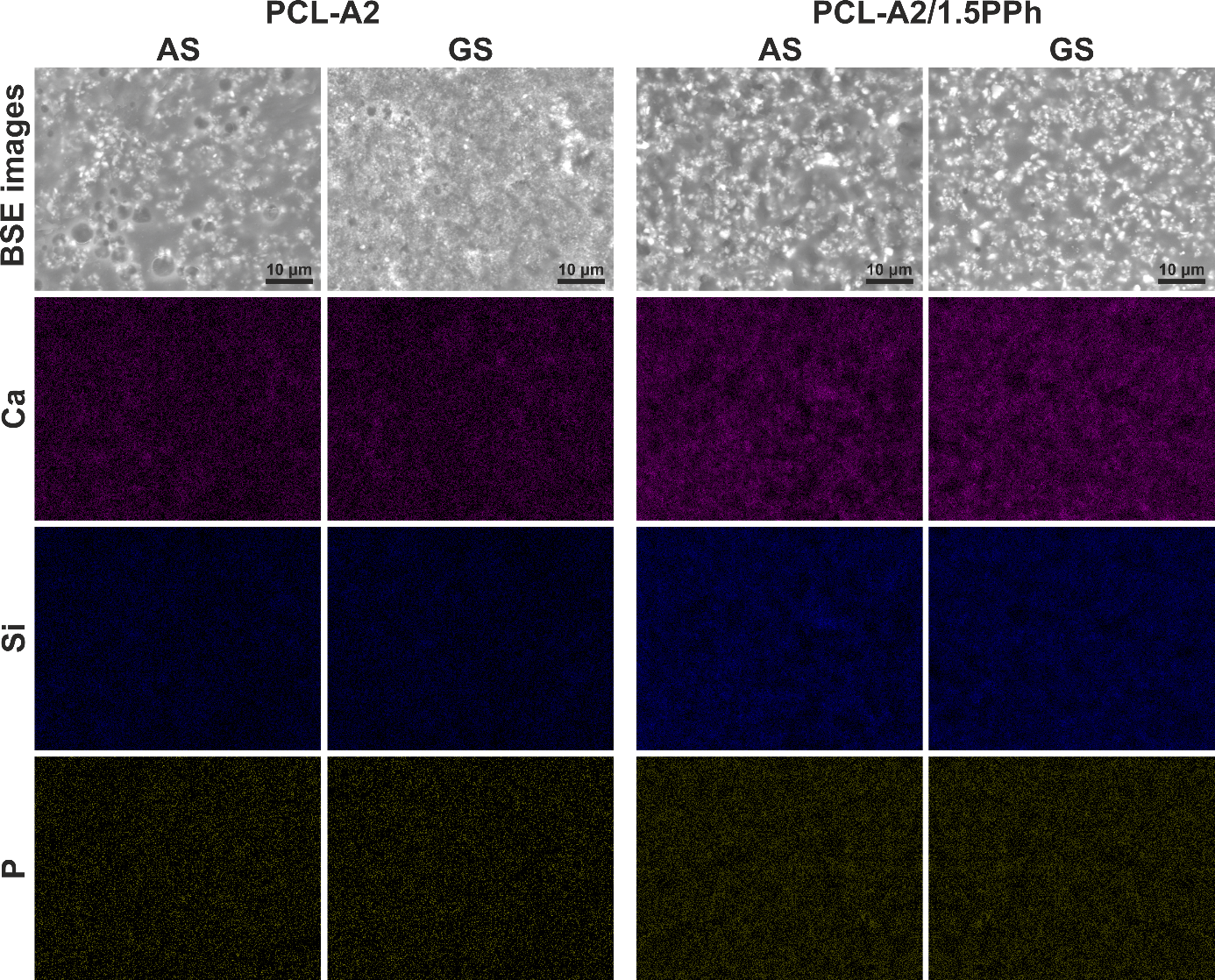


**Figure A.1.** BSE images and EDX elemental mapping of the AS and GS of the PCL-A2 and PCL-A2/1.5PPh films.


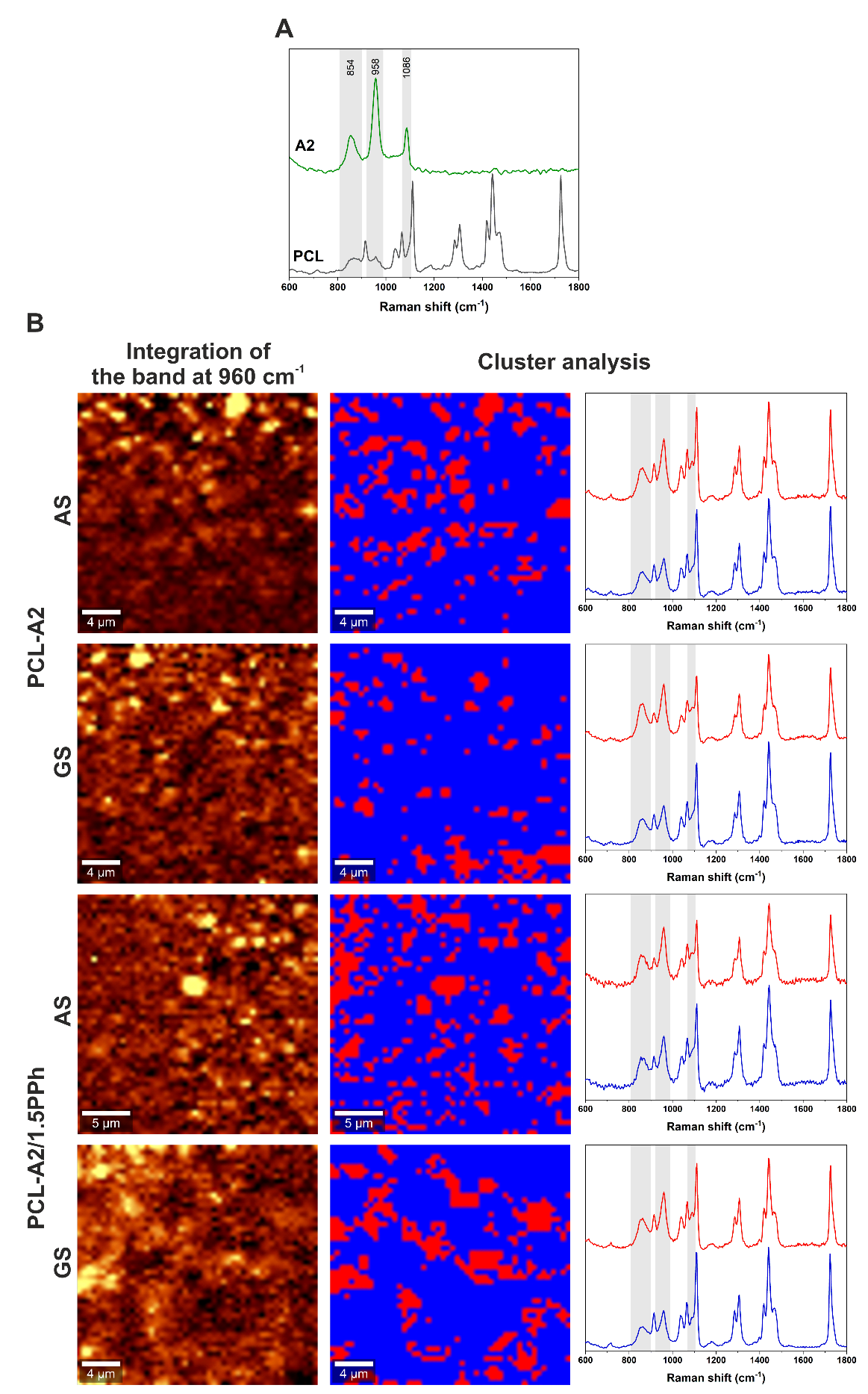


**Figure A.2.** Raman spectra of PCL film and bioactive glass (A). Raman images of the AS and GS of the PCL-A2 and PCL-A2/1.5PPh films showing distribution of BG particles (integration of the band at 958 cm^–1^), with CA images and averaged Raman spectra extracted from CA results (B). The colours of spectra correspond to the colours in CA images.





**Figure A.3.** ATR-FTIR spectra of AS and GS of the PCL/PPh films. For comparison FTIR spectrum of PPh extract is also shown.
